# Newly identified parathormone-secreting cells in the thyroid tissue

**DOI:** 10.1016/j.sopen.2026.06.004

**Published:** 2026-06-24

**Authors:** Özge Karabıyık Acar, Gamze Torun Kose, Ezgi Hacıhasanoglu, Alperen Tuncer, Gülnihal Bozdağ, Elif Yorgun, Fikrettin Şahin, Erhan Aysan

**Affiliations:** a Okan University, Faculty of Engineering and Natural Sciences, Department of Genetics and Bioengineering, Istanbul, Türkiye; b Yeditepe University, Faculty of Engineering, Department of Genetics and Bioengineering, Istanbul, Türkiye; c Yeditepe University, Faculty of Medicine, Department of Pathology, Istanbul, Türkiye; d Yeditepe University, Faculty of Medicine, Department of General Surgery, Division of Endocrine Surgery, Istanbul, Türkiye

**Keywords:** Parathormone, PTH, Thyroid, Parathyroid, Secretion, New cell

## Abstract

**Background:**

After total parathyroidectomy, circulating parathyroid hormone (PTH) levels typically do not fall to zero, and the biological source of this residual secretion remains unclear.

**Methods:**

Patients were divided into three groups. Group 1 (*n* = 46) comprised total thyroidectomy tissues; Group 2 (n = 46) included non-thyroid/non-parathyroid tissues (negative controls); and Group 3 (*n* = 7) consisted of healthy parathyroid gland tissues (positive controls). All tissues were kept in physiological saline immediately after resection for 30 min, and then 2 mL samples were taken to measure PTH concentrations. In addition, confocal immunofluorescence staining was performed on all specimens.

**Results:**

Mean PTH levels were 38.50 ± 7.92 pg/mL in Group 1, 6.95 ± 1.22 pg/mL in Group 2, and 648.00 ± 15.82 pg/mL in Group 3 (*p* = 0.01). Confocal microscopy after immunofluorescence staining revealed cells with PTH-positive granules in the thyroid tissue that were morphologically similar to thyroid epithelial cells.

**Conclusion:**

Thyroid tissues secrete a significant amount of PTH compared to many other tissues. This may be due to a new cell population identified by immunofluorescence staining.

## Highlights

• We first identified parathormone secretion from the thyroid gland. Subsequent immunohistological examinations revealed the presence of specialised cells within the thyroid gland that can secrete parathormone.

## Introduction

Parathormone (PTH) plays a central role in calcium–phosphate homeostasis, and its circulating level is primarily determined by the functional parathyroid glands. Theoretically, after total parathyroidectomy—particularly in patients with secondary hyperparathyroidism (SH), defined as excessive PTH secretion caused by chronic hypocalcaemic stimuli such as chronic kidney disease—circulating PTH levels are expected to fall to undetectable levels. However, numerous large clinical case series have demonstrated that this expected outcome is rarely achieved, and measurable serum PTH levels persist even after the apparently complete removal of all parathyroid glands [1], [2], [3], [4], [5], [6], [7].

This finding has traditionally been attributed to inadequate parathyroidectomy due to ectopic or super number parathyroid glands. While these factors may be involved in the etiology of some cases, they are not convincing due to their low incidence. Ectopic parathyroid glands are reported in less than 20% of cases, and süper number glands are much rarer [8], [9], [10]. Furthermore, serum PTH levels may remain detectable even in highly radical procedures involving thymusectomy and extensive cervical exploration.

Total thyroidectomy is indicated for patients with fine needle aspiration cytology results of Bethesda IV and V. Recent studies have reported that the risk of cancer is around 18–20% in patients with Bethesda III [11], and even in very rare cases, malignancy can occur in patients with Bethesda II [12]. Following carefully performed total thyroidectomy in which all four parathyroid glands are visualised and preserved with intact vascular supply, transient hypoparathyroidism and hypocalcaemia may still occur [13]. Although temporary ischaemia or surgical manipulation of the parathyroid glands is often considered responsible, these mechanisms do not fully explain all cases, particularly those in which the glands appear well perfused intraoperatively.

These two clinical observations—persistent measurable PTH after total parathyroidectomy and transient PTH reduction after thyroidectomy—suggest that additional, non-parathyroid gland sources of PTH or PTH-like secretion may exist.

Embryological and developmental evidence provides a theoretical basis for this possibility. The thyroid, parathyroid, and thymus glands originate from closely related pharyngeal pouch derivatives. Activation of the GCM2 gene in endodermal progenitor cells drives parathyroid differentiation, and during embryogenesis, thyroid and parathyroid tissues migrate in close proximity along the thyroglossal tract [14]. These developmental relationships raise the possibility that specialised cells with parathyroid-like functional properties may exist within thyroid tissue.

Based on these considerations, we hypothesised that thyroid tissue may contain cells capable of secreting PTH in small amounts. To test this hypothesis, we measured PTH levels in excised thyroid tissue specimens obtained during total thyroidectomy. Macroscopic and histopathological examinations were performed to exclude specimens containing inadvertently removed parathyroid tissue. PTH levels obtained from thyroid tissues were then compared with those from non-endocrine tissues (negative controls) and parathyroid tissues (positive controls). In addition, immunofluorescence staining was used to investigate the possible cellular source of PTH within thyroid tissue.

## Methods

The study protocol was reviewed and approved by the local clinical studies ethics committee (approval number: B.10.1.TKH.4.34.H.GP.0.01/252). The study was conducted in accordance with the ethical principles outlined in the Declaration of Helsinki and its most recent amendments. All surgical procedures were performed at Yeditepe University Hospital, Department of Endocrine Surgery. All patients were informed verbally and in writing about the purpose and procedures of the study prior to enrolment, and their questions were fully addressed.

A total of 92 patients (56 women and 36 men; female-to-male ratio: 1.55; age range: 18–61 years; median age: 47.5 years) who presented to our Endocrine Surgery clinic between February 2024 and September 2024 were included in the study. The patients were categorised into three groups:

• Group 1 (*n* = 46): patients undergoing total thyroidectomy
• Group 2 (n = 46): patients undergoing surgery unrelated to thyroid or parathyroid tissue (negative control)
• Group 3 (*n* = 7): cases from Group 1 in whom a parathyroid gland was either inadvertently excised or had to be excised due to capsular adherence (positive control).

Inclusion criteria for Group 1: age ≥ 18 years, no use of medications affecting calcium or PTH metabolism, normal preoperative serum calcium and PTH levels, indication for total thyroidectomy, and agreement to participate in the study.

Exclusion criteria for Group 1: age < 18 years, surgical procedures other than total thyroidectomy (e.g., lobectomy, subtotal thyroidectomy, or total thyroidectomy with any cervical lymph node dissection), or medications affecting serum calcium or PTH levels.

Inclusion criteria for Group 2: age ≥ 18 years, no use of medications affecting serum calcium or PTH levels, normal preoperative serum calcium and PTH levels, indication for a non-thyroid/non-parathyroid general surgery procedure, and agreement to participate.

Exclusion criteria for Group 2: age < 18 years, a very large amount of excised tissue that could interfere with standardisation, or medications affecting calcium/PTH levels.

Power analysis indicated that a minimum of 46 patients was required, with a Kruskal–Wallis test yielding 90% statistical power at a 95% confidence level, an effect size of 0.40, and a type I error rate < 0.05. A total of 99 cases were included in the final analysis. The number of cases in Group 3 (*n* = 7) reflects the total number of unintended parathyroid excisions during the study period.

All removed tissues were immediately washed with saline. Any remaining blood was removed by gently pressing the tissue. Each tissue was placed in a sterile container containing 50 mL of warm (36ᵒͦC) saline and left in the operating room for 30 min. At the end of this period, a 2 mL sample of the saline was taken and sent to the biochemistry laboratory for PTH measurement.

All tissues removed from Groups 1 and 2 were taken from the physiological saline solution and transferred to formalin-containing containers for routine histopathological examination. In Group 3, a small section of healthy parathyroid tissue was excised and transferred to formalin-containing containers for histopathological examination. The remaining parathyroid tissues in this group were autotransplanted into the neck muscles. Routine staining was performed on each tissue for histopathological diagnosis in the pathology laboratory. New sections were then cut from the tissues for immunofluorescence staining and confocal microscopic examination.

### Immunofluorescence staining

Monoclonal anti-parathyroid hormone antibody [rPTH/911] (Abcam®, ab234415; 1 μg/mL) was used as the primary antibody. For cytoskeletal staining, Alexa Fluor™ 546 Phalloidin (Invitrogen®; A22283; 1:1000), and for nuclear staining, DAPI (Sigma-Aldrich®; D8417; 1 μg/mL) were utilised. All samples were fixed in 10% neutral-buffered formalin for 2 h. Tissue processing was performed on the Leica ASP6025 Automated Vacuum Tissue Processor (Leica Biosystems®), after which formalin-fixed paraffin-embedded (FFPE) blocks were prepared.

Sections (4 μm thick) were incubated at 65 °C for 5 min, followed by washing with xylene three times for 10 min and absolute ethanol three times for 3 min. Sequential dehydration was then performed using 95%, 85%, and 75% ethanol solutions. Samples were permeabilised with 1% Triton X-100, blocked for 10 min, and washed with 1× PBS. Slides were incubated with the primary antibody for 1 h at room temperature (RT) and subsequently with the appropriate secondary antibody (Thermo Scientific®; 35,502; 1:1000) for 1 h at RT. DAPI staining was performed for 15 min to visualise nuclei. Finally, slides were washed in PBS, mounted in Aqua Poly/Mount, and examined using an LSM 700 confocal microscope (Carl Zeiss®, Germany).

## Statistical analysis

Descriptive statistics were expressed as percentages, frequencies, means, standard deviations, medians, and minimum–maximum values. The Kruskal–Wallis test was used to compare PTH measurements between the groups. Pairwise comparisons were performed using the Mann–Whitney *U* test with Bonferroni correction. Chi-square analysis was used for proportional comparisons between groups based on PTH cut-off levels. A *p*-value <0.05 was considered statistically significant. All statistical procedures were conducted using the SPSS 25.0 software package.

## Results

Histopathological examination of Group 1 revealed 19 (41.4%) papillary thyroid carcinoma cases, 4 (8.6%) micropapillary carcinoma cases, 21 (45.7%) benign multinodular goitre cases, and 2 (4.3%) solitary benign nodules. In Group 2, the excised tissues consisted of 15 (32.6%) neck skeletal muscle samples, 13 (29.2%) neck adipose tissue samples, 6 (13%) breast tissue samples, 6 (13%) axillary adipose tissue samples, 4 (8.6%) lipomas, 1 (2.1%) pancreatic tail segment, and 1 (2.1%) splenic tissue sample. Histopathological examination of all tissues in Group 3 revealed normal parathyroid tissue.

PTH measurements differed significantly among the groups (*p* = 0.01). The mean PTH level was 38.50 ± 32.92 pg/mL in Group 1, compared with 6.95 ± 1.22 pg/mL in Group 2 and 648.00 ± 553.82 pg/mL in Group 3 (Table 1).

**Table 1:** PTH values of the groups.

PTH Value (pg/mL)	Group 1 (*n* = 46)	Group 2 (n = 46)	Group 3 (n = 7)
<10		46 (100%) cases	–
10–99	43 (93,4%) cases	–	–
100–200	3 (6,6%) cases	–	–
>200	–	–	7 (100%) cases
Mean	38,50 ± 7,92 pg/mL	6,95 ± 1,22 pg/mL	648,00 ± 15,82 pg/mL
Range	37–114 pg/mL	5–9 pg/mL	220–1084 pg/mL

The distribution of PTH values also demonstrated clear differences between groups. In Group 1, 2 cases (4.3%) had PTH levels below 10 pg/mL, 41 cases (89.1%) had levels between 10 and 99 pg/mL, and 3 cases (6.6%) had levels between 100 and 200 pg/mL; no cases exceeded 200 pg/mL. In contrast, all samples in Group 2 (100%) had PTH levels below 10 pg/mL. In Group 3, all samples (100%) had PTH levels above 200 pg/mL. The observed ranges were 7–143 pg/mL in Group 1, 5–9 pg/mL in Group 2, and 220–1845 pg/mL in Group 3 (Table 1).

**Fig. 1:**
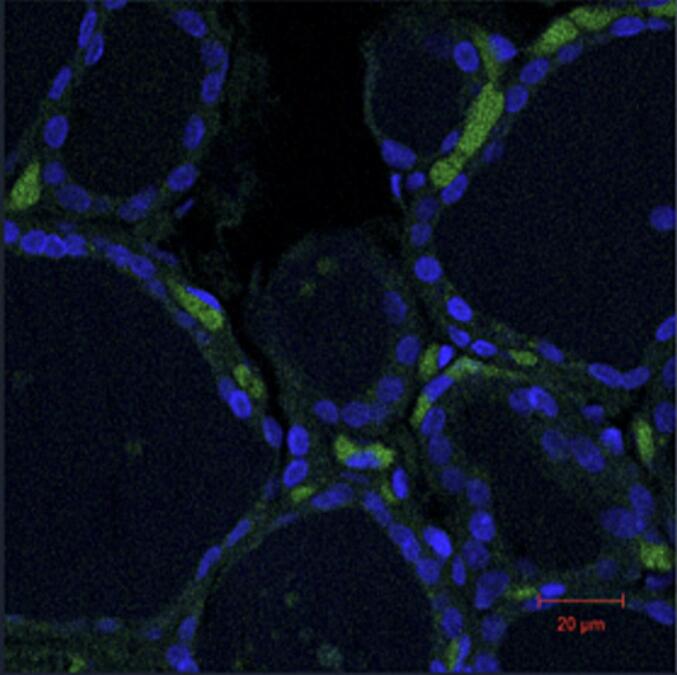
In confocal microscopic image, thyrocytes are seen around the thyroid follicles, with blue fluorescence (DAPI) stained nuclei. Among these cells, there are different cells of which their cytoplasms are stained with green fluorescence due to the PTH proteins. These are the PTH-secreted thyroid-like cells. (Scale bar 20μm)

**Fig. 2:**
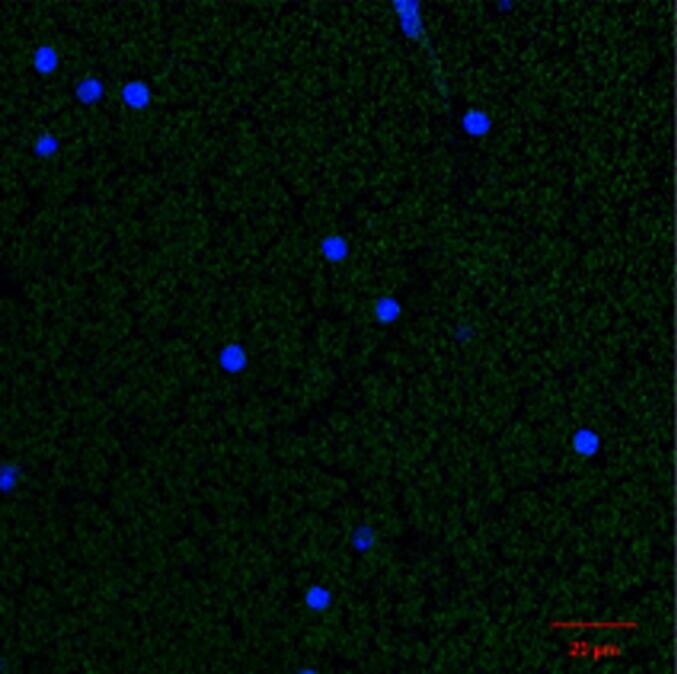
In confocal microscopic image, perithyroidal fatty tissue. Adipocytes are seen with blue fluorescence (DAPI) stained nuclei. (Scale bar 20μm)

**Fig. 3:**
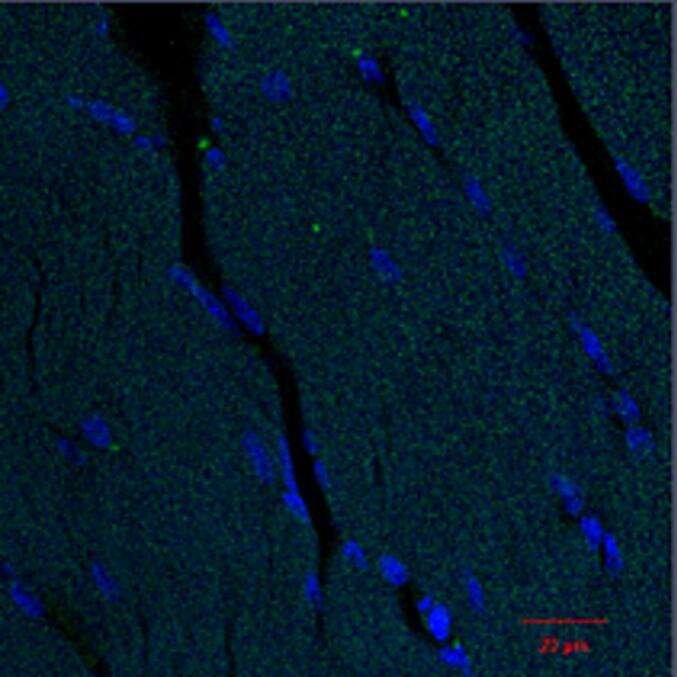
In confocal microscopic image, anterior neck region striated muscle tissue. Striated muscle cells are seen with blue fluorescence (DAPI) stained nuclei. (Scale bar 20μm)

**Fig. 4:**
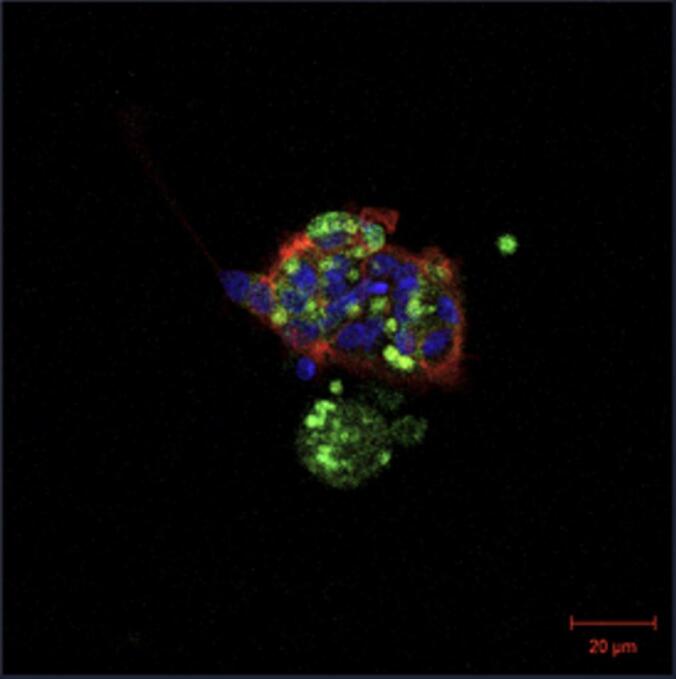
In confocal microscopic image, parathyroid tissue. Parathyroid chief cells are seen with blue fluorescence (DAPI) stained nuclei and green fluorescence stained cytoplasm. It is seen that the green fluorescent staining in the cytoplasm of these cells is much more intense compared to the PTH-secreted thyroid-like cells in Fig. 2. (Scale bar 20μm)

In immunofluorescence staining, some thyroid follicle cells in Group 1 had similar morphology to other follicle cells, but their cytoplasm contained granules stained with green fluorescent PTH. These cells were few in number within the thyroid parenchyma and were heterogeneously distributed. They were interpreted as PTH-secreting thyroid-like cells (Fig. 1). No cells with green fluorescent PTH-stained granules in their cytoplasm were detected in any tissue in Group 2 (Fig. 2, Fig. 3). In contrast, histopathological examination of parathyroid tissues in Group 3 revealed highly intense green fluorescent PTH-stained granules in the cytoplasm of all chief cells (Fig. 4).

**Fig. 5:**
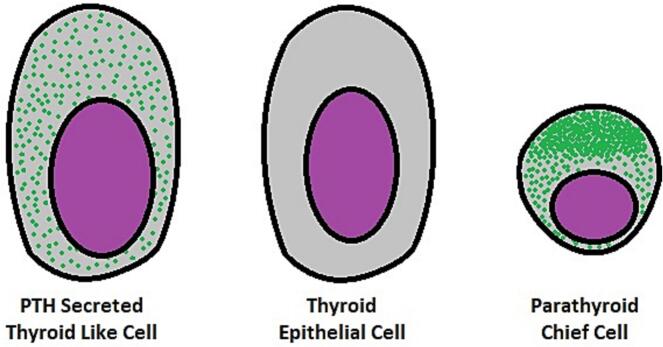
Illustration of PTH-secreted thyroid-like cell, thyroid epithelial cell and parathyroid chief cell.

Comparative morphologic analysis is illustrated in Fig. 5. The PTH-secreting thyroid-like cells (left) resemble thyrocytes in size and general architecture; however, the presence of distinct cytoplasmic fluorescent granules differentiates them. Chief cells demonstrate denser cytoplasmic granularity and are smaller and more spherical than both thyrocytes and PTH-secreting thyroid-like cells.

## Discussion

In the last few years, technological developments in the medical field have been rapid and are continuously evolving. One of the most revolutionary breakthroughs was the introduction of the internet of things concept within medical practice: Remote thyroid surgeries via telesurgery, consultations with thyroid specialists via telementoring, safe preoperative and postoperative patient monitoring are of great importance [15]. In the other hand 3D printing plays a significant and multifaceted role in improving healthcare services. Modeling of the thyroid and parathyroid glands with 3D printing has made important contributions to education, learning, project development, and the optimal planning of complex surgical procedures [16]. Despite these innovations, there are two serious unresolved problems in endocrine surgery: why serum PTH levels do not fall to zero after a successful total parathyroidectomy in SH patients, and why serum PTH slightly decreases even after a successful total thyroidectomy in which all parathyroid glands are preserved. These situations have traditionally been attributed to surgical technical factors such as incomplete resection in SH patients and ischaemia-related parathyroid dysfunction in total thyroidectomy patients [13], [17], [18].

Zhou et al. reported a mean postoperative serum PTH level of 13.4 pg/mL in 55 SH patients six months after total parathyroidectomy [4]. Bi et al. found postoperative PTH levels of a mean of 11.5 (10.8–24.5) pg/mL in patients with severe hypocalcaemia and a mean of 10.9 (10.1–23.8) pg/mL in those without severe hypocalcaemia after total parathyroidectomy [5]. Iorga et al. observed a lowest postoperative level with a mean of 4.5 pg/mL even after excluding cases of recurrence or persistence [6]. Similarly, Xixiang et al. reported that mean PTH levels, which dropped to 9.6 pg/mL on postoperative day one but increased to 17.8 pg/mL at 6 months and 43.3 pg/mL at one year [7]. Liang et al. showed that the mean serum PTH level decreased from 1985 pg/mL preoperatively to a mean of 54 pg/mL on postoperative day one, followed by a rise to a mean of 65 pg/mL at one month [15]. Total parathyroidectomy was performed in all of the above studies, and none involved autotransplantation.

These data clearly show that serum PTH does not disappear after total parathyroidectomy and instead shows a gradual increase over time. Despite this remarkable finding, very few studies in the literature have attempted to explain this phenomenon. Aly et al. hypothesised that microscopic embryological remnants of parathyroid tissue in the thyroid, thymus, or cervical fat may become activated under physiological stress such as renal failure or total parathyroidectomy [17].

Supporting this, Burgstaller et al. performed an extremely radical surgical protocol in 109 SH cases, including bilateral neck exploration, resection of all visible parathyroids, bilateral transcervical thymectomy, and extensive microdissection of central cervical adipose tissue [19]. Even after this radical operation, serum PTH remained between 10 and 65 pg/mL in 22% of cases and above 65 pg/mL in 3%. The article did not report whether “zero level” PTH was actually achieved, suggesting that complete absence of PTH may not be achieved.

We believe that the hypothesis of Aly et al. is important [17]. The parathyroid, thyroid, and thymus are endocrine organs with a common embryological origin [14]. There may be specialised embryological rests in these organs that perform the functions of each other, or stem cells that have the potential to undergo transformation.

In this study, we focused on the possibility of PTH-secreting specialised cells in thyroid tissue. Indeed, in the study by Burgstaller et al., despite total parathyroidectomy, thymectomy, and extensive cervical fatty/lymphatic tissue excision, the fact that PTH did not reach zero supports our hypothesis. In our opinion, the presence of thyroid tissue may be one of the reasons why PTH does not decrease to the expected level in these cases.

We compared the amount of PTH secreted from thyroid tissue with that from non-thyroid and non-parathyroid tissues (negative control group) and demonstrated that thyroid tissue secretes more PTH than these tissues. In this negative control group, we primarily chose perithyroidal fatty tissues and strap muscles in order to prevent falsely elevated PTH levels that might be present in venous blood in this region. On the other hand, we performed the same measurements on healthy parathyroid glands as a positive control group. Of course, these parathyroid glands were not excised for the purpose of PTH measurement. We are an endocrine surgery clinic that performs a high volume of thyroid surgery. Although rare, some parathyroid glands are accidentally excised during total thyroidectomy or are attached to the thyroid capsule and receive their vascular supply directly from the thyroid capsule. These glands are necessarily excised. Such glands are kept in physiological saline during surgery and are cut into thin slices before the surgery is completed and autotransplanted into the strap muscles. Here, we kept these tissues in physiological saline for a standard period defined in this research (30 min) and measured PTH in this fluid before autotransplantation.

In this study, we not only biochemically demonstrated the secretion of PTH from thyroid tissue, but also aimed to identify the source of this secretion. The source could be a new type of cell. Since the PTH values from thyroid tissues showed significant differences (37 pg/mL vs. 114 pg/mL), we considered that the PTH-secreting cells might show heterogeneous distribution within the tissue and that it would be difficult to detect them.

As seen in Fig. 1, we detected new cells located among the thyrocytes in the natural thyroid follicle structure. Their morphology was slightly different from thyrocytes, and their cytoplasm was PTH-positive. These cells are not ectopic intrathyroidal parathyroid glands because they are not clustered together like intrathyroidal parathyroid glands and are not separated from the surrounding cells by a fibrous capsule. These cells are located within the natural thyroid follicle structure. In addition, the morphology of these cells is different from the spherical structure of parathyroid chief cells; they have a more prismatic structure and are very similar to thyrocytes. These morphological data suggest that they may represent a different cell type.

The microanatomy of these cells is similar to calcitonin-secreting parafollicular cells. As is known, parafollicular cells are located within the follicular structure and do not show characteristics of grouping or encapsulation. They are found as single cells in thyroid tissue, and their distribution is heterogeneous. Since the morphology of these cells differs from thyrocytes (slightly larger and C-shaped), they are easily detected in routine histopathological evaluations. However, the morphology of the intrathyroidal PTH-secreting specialised cells detected here is very similar to thyrocytes, and they could only be detected with immunofluorescent PTH staining.

The data presented here may help to explain why blood PTH does not fall to the expected zero level after total parathyroidectomy in SH cases. Our data may also help to explain the slight decrease in serum PTH levels and transient hypocalcaemia following thyroid surgeries in which the parathyroid glands are successfully preserved. The heterogeneity of outcomes after these surgeries (in some SH cases PTH decreases significantly, while in others it remains relatively high; in some successful total thyroidectomies PTH decreases slightly, while in others it decreases severely enough to cause symptomatic transient hypocalcaemia) may be related to the numerical and functional heterogeneity of specialised PTH-secreting cells in thyroid tissue.

## CRediT authorship contribution statement

**Özge Karabıyık Acar:** Investigation, Data curation, Conceptualization. **Gamze Torun Kose:** Writing – original draft, Data curation, Conceptualization. **Ezgi Hacıhasanoglu:** Writing – original draft, Data curation, Conceptualization. **Alperen Tuncer:** Methodology, Investigation, Data curation. **Gülnihal Bozdağ:** Writing – review & editing, Writing – original draft, Data curation. **Elif Yorgun:** Writing – original draft, Data curation. **Fikrettin Şahin:** Visualization, Supervision, Data curation. **Erhan Aysan:** Writing – original draft, Data curation, Conceptualization.

## Ethics approval

The study protocol was reviewed and approved by the local clinical studies ethics committee (approval number: B.10.1.TKH.4.34.H.GP.0.01/252).

## Funding sources

This research did not receive any specific grant from funding agencies in the public, commercial, or not-for-profit sectors.

## Declaration of competing interest

The authors declare that they have no known competing financial interests or personal relationships that could have appeared to influence the work reported in this paper.
